# A Framework Integrating GWAS and Genomic Selection to Enhance Prediction Accuracy of Economical Traits in Common Carp

**DOI:** 10.3390/ijms26147009

**Published:** 2025-07-21

**Authors:** Zhipeng Sun, Yuhan Fu, Xiaoyue Zhu, Ruixin Zhang, Yongjun Shu, Xianhu Zheng, Guo Hu

**Affiliations:** 1 Key Laboratory of Freshwater Aquatic Biotechnology and Breeding, Ministry of Agriculture and Rural Affairs, Heilongjiang River Fisheries Research Institute, Chinese Academy of Fishery Sciences, Harbin 150070, China; sunzhipeng@hrfri.ac.cn (Z.S.); fuyuhan2000@outlook.com (Y.F.); zhuxiaoyue2001@126.com (X.Z.); zrxin2001@126.com (R.Z.); 2 Key Laboratory of Molecular Cytogenetics and Genetic Breeding of Heilongjiang Province, College of Life Science and Technology, Harbin Normal University, Harbin 150025, China; shuyj@hrbnu.edu.cn

**Keywords:** common carp, GWAS, GS, FarmCPU, heritability

## Abstract

Common carp (*Cyprinus carpio*) is one of the most significant fish species worldwide, with its natural distribution spanning Europe and Asia. To conduct a genome-wide association study (GWAS) and compare the prediction accuracy of genomic selection (GS) models for the growth traits of common carp in spring and autumn at 2 years of age, a total of 325 carp individuals were re-sequenced and phenotypic measurements were taken. Three GWAS methods (FarmCPU, GEMMA, and GLM) were applied and their performance was evaluated in conjunction with various GS models, using significance levels based on *p*-values. GWAS analyses were performed on eight traits (including the body length, body weight, fat content of fillet, and condition factor) for both spring and autumn seasons. Eleven different GS models (such as Bayes A, Bayes B, and SVR-linear) were combined to evaluate their performance in genomic selection. The results demonstrate that the FarmCPU method consistently exhibits superior stability and predictive accuracy across most traits, particularly under higher SNP densities (e.g., 5K), where prediction accuracies frequently exceed 0.8. Notably, when integrated with Bayesian approaches, FarmCPU achieves a substantial performance boost, with the prediction accuracy reaching as high as 0.95 for the autumn body weight, highlighting its potential for high-resolution genomic prediction. In contrast, GEMMA and GLM exhibited a more variable performance at lower SNP densities. Overall, the integration of FarmCPU with genomic selection (GS) models offers one of the most reliable and efficient frameworks for trait prediction, particularly for complex traits with substantial genetic variation. This approach proves especially powerful when coupled with Bayesian methodologies, further enhancing its applicability in advanced breeding programs.

## 1. Introduction

The common carp (*Cyprinus carpio*) is a widely distributed and economically significant freshwater fish species with a domestication history of over two thousand years [1]. Native to Eastern Europe and Asia, it has been introduced globally due to its strong adaptability and high market demand. Common carp plays a crucial role in aquaculture because of its rapid growth rate, ability to adapt to various environmental conditions, and its significant impact on economic livelihoods as a food source [2].

With the continuous advancement of genetic technologies, the application of genome-wide association studies (GWAS) and genomic selection (GS) methods in animal genetic breeding has gradually become a research hotspot, particularly in the field of aquaculture. As modern breeding technologies, they have been widely applied in livestock and plant breeding, significantly improving the accuracy and efficiency of trait selection through high-density molecular markers. In recent years, the rapid development of high-throughput sequencing and molecular breeding technologies, along with the completion of high-quality genomes for numerous aquatic species, has made it possible to conduct trait analysis and breeding value prediction at the whole-genome level for aquatic organisms. Aquaculture breeding in China is transitioning from traditional breeding techniques, such as selective breeding, hybrid breeding, and polyploid manipulation, to modern molecular breeding technologies. Since the completion of the genome sequencing of the oyster (*Crassostrea gigas*) in 2020 [3], genome mapping has been completed for more than 50 aquaculture species, including the half-smooth tongue sole (*Cynoglossus semilaevis*) [4], common carp (*Cyprinus carpio*) [5], large yellow croaker (*Larimichthys crocea*) [6], rainbow trout (*Oncorhynchus mykiss*) [7], and Atlantic salmon (*Salmo salar*) [8].

Genomic Selection (GS) is a modern breeding method that uses high-density genetic markers to predict an individual’s breeding value, significantly enhancing the accuracy and efficiency of selection. Unlike traditional breeding, which relies on phenotypic observations, GS allows for selection based on the genetic potential of an individual, even before the traits are expressed. GS has been widely applied in livestock and plant breeding, significantly accelerating the improvement of traits such as growth, disease resistance, and yield. In aquaculture species, GS has surpassed traditional pedigree-based selection and has been successfully applied to the improvement of traits such as growth and disease resistance, as seen in Atlantic salmon (*Salmo salar*) [9], catfish (*Clarias batrachus*) [10], and gilthead seabream (*Sparus aurata*) [11]. GWAS methods are widely used to identify genetic markers associated with economic traits. In aquaculture, Palaiokostas et al. (2018) conducted a GWAS analysis on the juvenile growth rate in common carp using RAD-seq technology and performed mixed linear model analysis with the R/gaston software [12]. They found that GS significantly outperformed traditional pedigree-based prediction methods in improving the breeding value prediction accuracy. In another study, Zheng et al. (2016) conducted a GWAS on fat traits in common carp using a 250K SNP chip and performed the association analysis with GEMMA software [13]. They identified several candidate genes and demonstrated the potential of GS for genetic improvement [13]. These advancements highlight the immense potential of GS in accelerating genetic the improvement of common carp and promoting sustainable aquaculture. As an important aquatic resource, the efficiency of genetic improvement in fish is crucial for enhancing productivity and disease resistance. However, in practical applications, there are significant differences in the performance of various combinations of GWAS models and GS models [8,14]. Therefore, exploring and optimizing the combined use of these methods is of paramount importance.

This study aims to analyze the impact of GS on four important traits (body length: SL, body weight: Weight, fat content of the fillet: FC, and the condition factor: CF) in spring and autumn seasons under different SNP densities through the combined application of various GWAS methods (FarmCPU, GEMMA, and GLM) with 11 different GS models. Special attention is given to the performance of FarmCPU, GEMMA, and GLM when integrated with GS models, exploring the differences in their stability and accuracy within genomic selection. By comparing the genomic selection performance of different method combinations, this study provides more accurate tools for genetic improvement in fish and offers a reference for future genomic selection research. The core objective of this study is to evaluate the optimization of fish genetic selection through the combined use of GWAS and GS methods, further investigating the performance differences of these methods across seasonal variations in spring and autumn and proposing the most suitable combination strategies for practical breeding applications. This not only helps to enhance the efficiency of genomic selection, but also provides scientific support for the genetic improvement of aquaculture.

## 2. Results

### 2.1. Phenotypic Analysis

**Figure 1:**
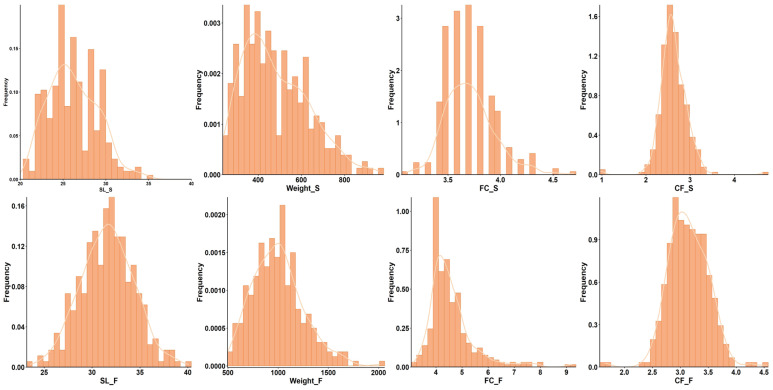
Frequency distribution histograms of the eight phenotypes. SL represents body length, Weight represents body weight, FC refers to the fat content of the fillet, CF stands for condition factor, and S and F refer to spring and autumn, respectively.

This study analyzes the body length (SL), body weight (Weight), fat content of the fillet (FC), and condition factor (CF) of carp in both spring (S) and autumn (F) seasons, using a detailed visual analysis through frequency density curves. The distribution of each phenotype is shown in the Figure 1. The spring season traits showed a more pronounced peak compared to autumn: the spring body length (SL_S) was distributed in a narrow range, concentrated between 20–35 cm, with an average of about 30 cm, while the autumn body length (SL_F) showed a broader distribution, ranging from 25–40 cm, with an average of about 32 cm. The spring body weight (Weight_S) showed a skewed distribution, concentrated between 400–800 g, with the highest value approaching 1000 g, whereas the autumn body weight ranged from 500 to 2000. The spring fat content of the fillet (FC_S) ranged between 3.5–4.5%, with an average of 4%, while the autumn fat (FC_F) concentrated between 4 and 6%, with a mean of about 5%, peaking at 6%. The condition factor (CF_S) in spring was concentrated between 2 and 4, with an average of about 3.5, while autumn showed a higher concentration at 4.5.

**Figure 2:**
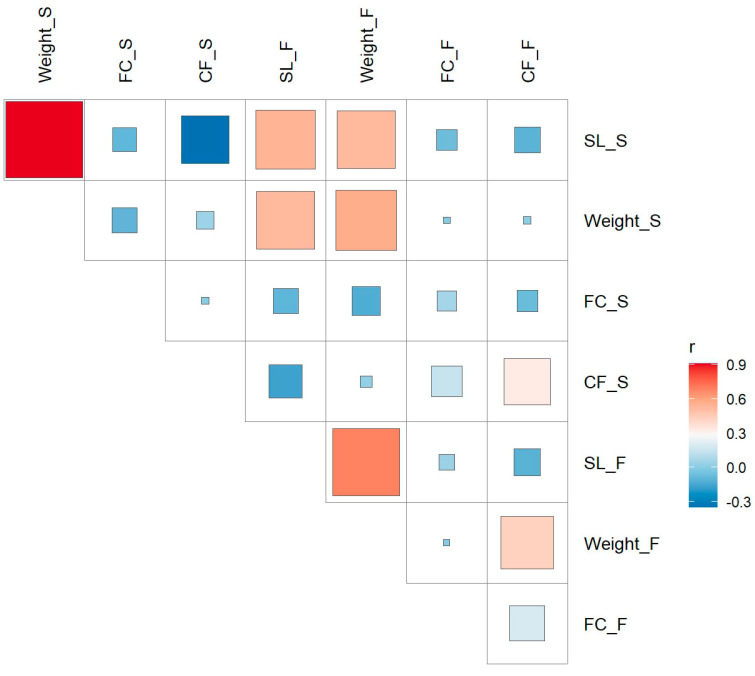
Phenotypic correlation heatmap between different traits in spring and autumn. Each cell shows the Pearson correlation coefficient (r value) between two traits, with the color representing the strength and direction of the correlation. Red indicates a positive correlation, blue indicates a negative correlation, and darker colors represent stronger correlations. The larger boxes highlight groups of traits with particularly strong correlations (|r| > [threshold value. The traits in the figure include body length (SL), body weight (Weight), fat content of the fillet (FC), and condition factor (CF), where “S” refers to spring and “F” refers to autumn.

Overall, the phenotypic data suggest that spring traits (SL_S, Weight_S, and FC_S) are more tightly distributed, which might be related to environmental conditions such as warmer temperatures or an increased energy demand. The Figure 2 correlations indicate strong positive correlations between the spring body length (SL_S) and weight (Weight_S) (r ≈ 0.9), body weight (Weight), and condition factor (CF) (r ≈ 0.6), and weak correlations between the fat content of the fillet (FC_S and FC_F) and the condition factor (CF_S and CF_F) (r ≈ 0.3). This reflects that the body length and body weight show a higher degree of seasonal consistency, while the fat content of the fillet and condition factor are more sensitive to seasonal changes.

### 2.2. Seasonal Heritability of Growth Traits via fastGWA-REML

As shown in Table 1, based on the fastGWA-REML method for single-nucleotide polymorphism (SNP) analysis, this study assessed the heritability, genetic variance (Vg), and environmental variance (Ve) of the body length (SL), body weight (Weight), fat content of the fillet (FC), and condition factor (CF) in a species during the spring (S) and autumn (F) seasons. The results reveal the impact of seasonal variation on the genetic contribution of traits. The findings show that the body length (SL_S 33.67%, SL_F 52.98%) and fat content of the fillet (FC_F 54.26%) had significantly higher genetic contributions in autumn compared to spring, while the body weight (Weight_S 31.45%, Weight_F 35.34%) showed minimal differences in genetic contributions between the two seasons. The genetic contribution of the fat content of the fillet (FC_S) in spring was abnormally low (6.17 × 10^−17^), which may be due to the strong influence of environmental factors on this trait, resulting in a limited genetic variation. Similarly, the heritability of the condition factor (CF_F) was also low (0.0234), suggesting that environmental factors may have a substantial impact on this trait as well, limiting the genetic influence.

A comparison of the genetic variance (Vg) and environmental variance (Ve) indicates that the body weight and condition factor are primarily influenced by environmental factors, while the body length and fat content of the fillet exhibit enhanced genetic effects in autumn. This suggests that the autumn environment may facilitate the expression of genetic factors, making it more suitable for genetic selection studies. Overall, the heritability of the body length and fat content of the fillet in autumn is significantly higher than in spring, with an increased proportion of genetic variance, suggesting that the autumn environment may enhance the expression of genetic factors. In contrast, the body weight and condition factor are primarily influenced by the environment in both seasons, with the condition factor having a very low genetic contribution in autumn, indicating that environmental factors (such as food and temperature) play a more significant role in its regulation. This suggests that traits like the condition factor can be optimized through environmental management, while traits with high heritability (such as SL_F and FC_F) may be suitable for genetic selection breeding studies.

### 2.3. Comparison of Significant SNP Detection Results for Growth Traits Across Spring and Autumn Seasons Using Three GWAS Methods

**Figure 3:**
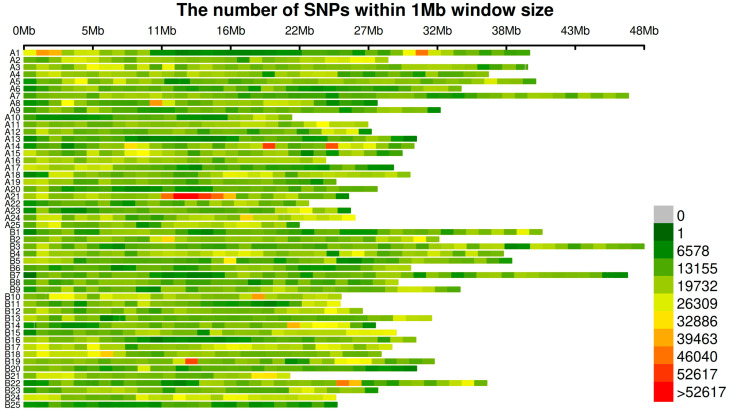
The figure shows the number of SNPs within a 1 Mb window size across different chromosomal regions. Each row represents a distinct chromosomal region, with the X-axis indicating different chromosomal intervals (from 0 Mb to 48 Mb). The color gradient reflects the number of SNPs within each interval, ranging from green (low SNP count) to red (high SNP count). The figure highlights the distribution of SNPs across the genome, identifying regions with higher SNP density. The color scale on the right indicates the corresponding SNP count range for each color.

**Figure 4:**
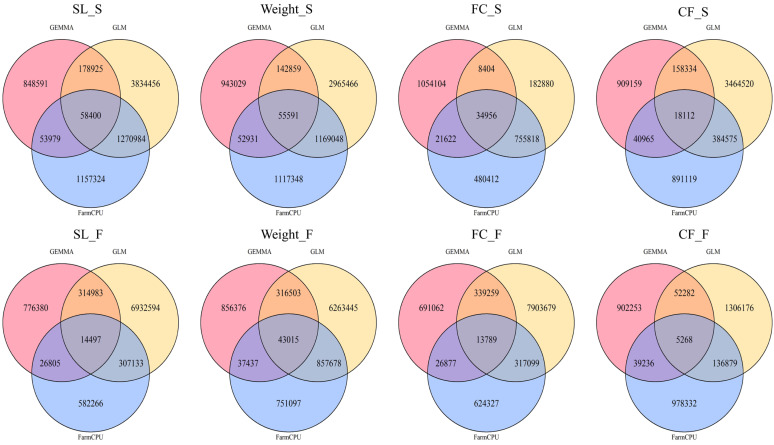
Overlap of significant SNPs identified by three GWAS methods for four traits across two seasons. The number of SNPs with *p*-values less than 0.05 detected by three GWAS methods (GEMMA in pink, GLM in yellow, and FarmCPU in blue) is shown using Venn diagrams. The traits include body length (SL), body weight (Weight), fillet fat content (FC), and condition factor (CF), with “S” representing spring and “F” representing autumn.

The SNP density on each chromosome was visualized, as shown in Figure 3. To compare the detection capabilities of the three GWAS methods—GEMMA, GLM, and FarmCPU—across different seasons and traits, the number of significant single nucleotide polymorphisms (SNPs) for four traits (the body length (SL), body weight (Weight), fat content of the fillet (FC), and condition factor (CF)) in the spring (S) and autumn (F) seasons was analyzed. A significance threshold of a *p*-value < 0.05 was applied for all analyses and the results are presented in a Venn diagram (Figure 4). Each diagram for the traits and seasons illustrates the number of SNPs detected by the three methods, along with their intersections and differences.

GLM detected the largest number of SNPs and showed a considerable overlap with the results from FarmCPU. This indicates that the GLM method performs strongly in SNP detection for spring traits, although it may also result in more false positives. FarmCPU also detected significant SNPs for these traits, with good overlap with GLM results, suggesting its advantage in complementing and refining the results from GLM. Overall, GLM detected the most SNPs across most traits, with a large overlap with FarmCPU, demonstrating its strong SNP detection ability. However, FarmCPU provided more stable and consistent results, particularly excelling in handling false positives.

### 2.4. Comparison of the Distribution of Significant SNP β Effect Sizes Across Different GWAS Methods

**Figure 5:**
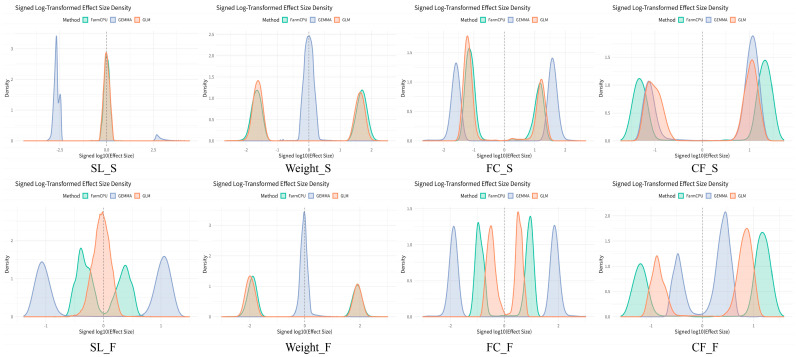
Density distribution of log-transformed effect sizes for significant SNPs detected by three GWAS methods. The figure shows the distribution of signed log_10_-transformed β effect sizes for SNPs with *p*-values less than 0.05, identified using FarmCPU (green), GEMMA (purple), and GLM (orange). Traits include body length (SL), body weight (Weight), fillet fat content (FC), and condition factor (CF), with “S” indicating spring and “F” indicating autumn. The plots highlight differences in effect size distributions across methods for each trait and season.

To explore the stability and consistency of different GWAS methods in estimating effect sizes, the performance of each method on different traits was compared. As shown in Figure 5, this plot presents the density distribution of β effect sizes for significant SNPs (*p*-value < 0.05) across eight traits, as estimated by each GWAS method (GEMMA, GLM, and FarmCPU). Each subplot represents the distribution of β effect sizes for different traits (body length (SL), body weight (Weight), fat content of the fillet (FC), and condition factor (CF)) in both spring (S) and autumn (F). The x-axis represents the signed log(β effect size), while the y-axis shows the corresponding density.

From the figure, it can be seen that the β effect size distribution for FarmCPU is more concentrated, indicating more consistent effect size estimates. In most traits, the distribution of the β effect sizes for FarmCPU is relatively narrower, suggesting that it provides more stable and reliable estimates. In contrast, the β effect size distributions for GEMMA and GLM are broader, especially for the body length (SL) and fat content of the fillet (FC), where the distributions are more dispersed, indicating poorer consistency in the effect size estimation for these methods. Notably, for the body length (SL) and body weight (Weight), the β effect size distributions of GEMMA and GLM are quite similar, showing overlapping patterns, which suggests these two methods exhibit greater consistency in effect size estimation for these traits. In contrast, FarmCPU’s β effect size distribution for these traits is more concentrated, demonstrating higher stability.

For the fat content of the fillet (FC) and condition factor (CF), the β effect size distribution of FarmCPU is more distinct compared to the other two methods (GEMMA and GLM) and tends to be skewed towards smaller effect sizes, which may indicate that FarmCPU provides more conservative and stable estimates for these traits. Overall, FarmCPU shows a clear advantage in the stability and consistency of effect size estimates, especially for the body length (SL), body weight (Weight), and fat content of the fillet (FC). Its β effect size distributions are more concentrated, suggesting that FarmCPU may provide more reliable results in the genetic analysis of these traits.

### 2.5. Comparison of Genomic Selection Performance of Different GWAS Methods and GS Models at Different SNP Densities

**Figure 6:**
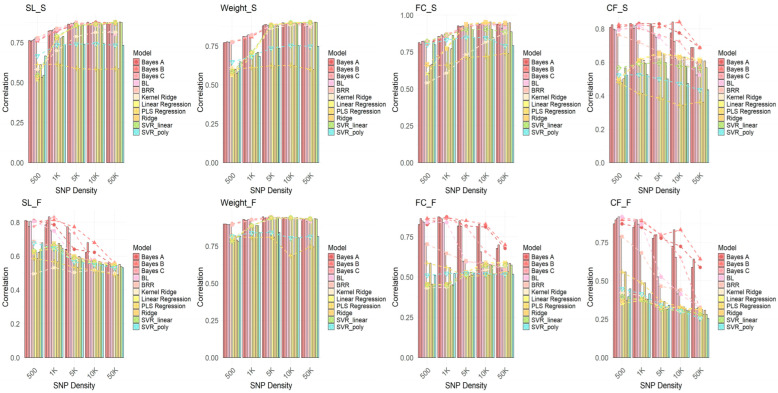
Results of genomic selection across 11 models following GWAS analysis using FarmCPU. The plot shows the correlation results for SNPs selected based on *p*-values, with SNP densities of 500, 1K, 5K, 10K, and 50K. Each model is represented by a different color, illustrating the performance of genomic selection at various SNP densities for different models.Traits include body length (SL), body weight (Weight), fillet fat content (FC), and condition factor (CF), with “S” indicating spring and “F” indicating autumn.

**Figure 7:**
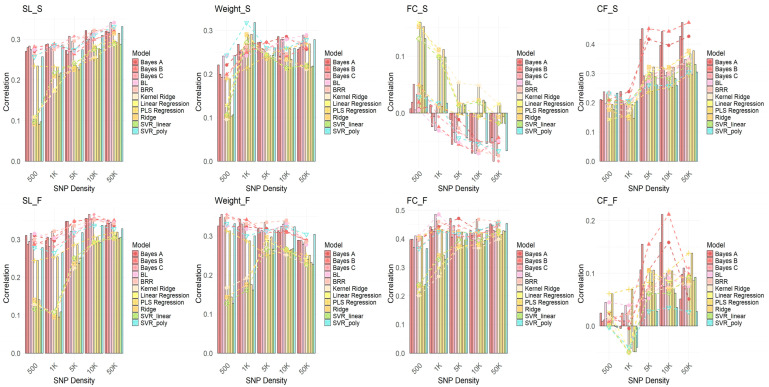
Results of genomic selection across 11 models following GWAS analysis using GEMMA. The plot shows the correlation results for SNPs selected based on *p*-values, with SNP densities of 500, 1K, 5K, 10K, and 50K. Each model is represented by a different color, illustrating the performance of genomic selection at various SNP densities for different models. Traits include body length (SL), body weight (Weight), fillet fat content (FC), and condition factor (CF), with “S” indicating spring and “F” indicating autumn.

**Figure 8:**
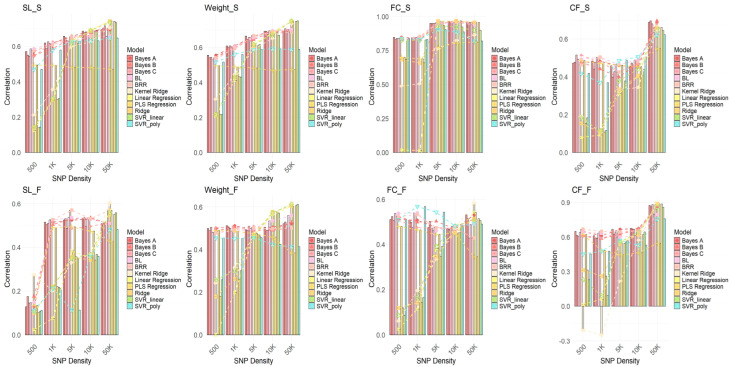
Results of genomic selection across 11 models following GWAS analysis using GLM. The plot shows the correlation results for SNPs selected based on *p*-values, with SNP densities of 500, 1K, 5K, 10K, and 50K. Each model is represented by a different color, illustrating the performance of genomic selection at various SNP densities for different models. Traits include body length (SL), body weight (Weight), fillet fat content (FC), and condition factor (CF), with “S” indicating spring and “F” indicating autumn.

To investigate the performance of different GWAS methods in genomic selection (GS) and to explore the impact of varying SNP densities on model performance, GWAS analyses were conducted using each GWAS model (FarmCPU, GEMMA, and GLM). SNPs were selected at densities of 500, 1K, 5K, 10K, and 50K, and genomic selection was performed using 11 GS models. The correlation of each model’s performance at different SNP densities was evaluated. As shown in Figure 6, Figure 7 and Figure 8, the results demonstrate that FarmCPU outperforms the other two methods (GEMMA and GLM) at all SNP densities, especially at higher SNP densities (e.g., 10K and 50K), where FarmCPU, in combination with various GS models, exhibited a strong and stable correlation. The prediction accuracy consistently exceeded 0.75, with some traits reaching above 0.9. In contrast, GEMMA and GLM showed lower correlations at lower SNP densities (e.g., 500 SNPs), with their performance exhibiting considerable fluctuation across different SNP densities, particularly for traits like the fat content of the fillet (FC) and condition factor (CF), where the correlations were weak. This suggests that GEMMA and GLM have a less stable genomic selection performance at lower SNP densities compared to FarmCPU.

In conclusion, the FarmCPU method demonstrates higher stability and consistency in genomic selection analysis, particularly under higher SNP density conditions, providing more reliable selection results. In contrast, GEMMA and GLM perform poorly at lower SNP densities. Therefore, FarmCPU is the recommended method for genomic selection applications.

### 2.6. Prediction Accuracy of GWAS-GS Models for Seasonal Traits Using 5K SNPs

**Figure 9:**
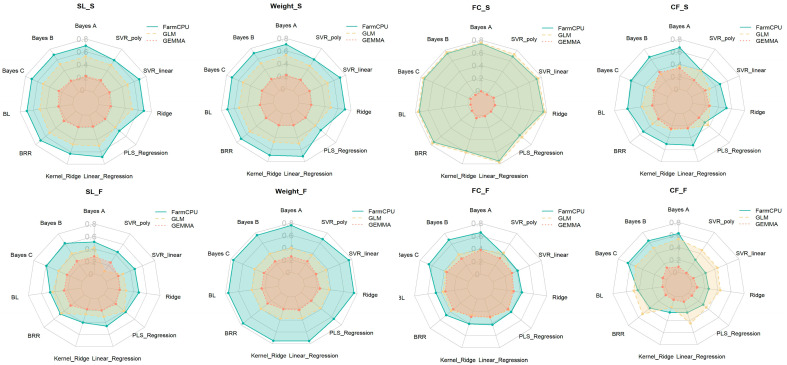
Analysis results of different GWAS and genomic selection (GS) model combinations using 5K SNPs. The radar charts illustrate the performance of 11 different models across eight traits (SL_S, Weight_S, FC_S, CF_S, SL_F, Weight_F, FC_F, and CF_F) based on the selected 5000 SNPs. The three methods (FarmCPU, GLM, and GEMMA) are compared to visualize the correlations across each model’s performance.

To further analyze the impact of combining genomic selection (GS) models with the three GWAS methods (FarmCPU, GEMMA, and GLM) on the genomic selection performance, the combination of these GWAS methods with 11 GS models (including Bayes A, Bayes B, Bayes C, BL, BRR, Kernel Ridge, Linear Regression, PLS Regression, Ridge, SVR linear, and SVR poly) was evaluated under the condition of selecting 5000 single-nucleotide polymorphisms (SNPs). As shown in Figure 9, the prediction accuracy for four traits (body length (SL), body weight (Weight), fat content of the fillet (FC), and condition factor (CF)) in both spring (S) and autumn (F) is presented. The results indicate that the combination of the FarmCPU method with multiple genomic selection (GS) models, such as Bayes A, Bayes B, and SVR_linear, consistently delivers the most stable and accurate performance across most traits. This approach shows a particularly high correlation at elevated SNP densities (e.g., 5K), providing reliable selection outcomes. Notably, for the FC_S trait, the prediction accuracy reaches up to 0.95, highlighting its effectiveness in the genomic prediction. In contrast, the combination of GEMMA with certain GS models (such as Kernel_Ridge and Ridge) performed relatively weakly for some traits, especially for the fat content of the fillet (FC), where its performance was inferior to that of FarmCPU. Furthermore, the performance of the GLM method was more variable, particularly at lower SNP densities, where its combination with some GS models showed poor results. Overall, the combination of FarmCPU with GS models provided the most stable and efficient results for genomic selection, particularly for traits with more complex genetic variation.

## 3. Discussion

### 3.1. Performance Differences Across GWAS Methods and the Impact of Algorithms

The results of this study demonstrated that FarmCPU consistently outperformed GEMMA and GLM across all traits and seasons, particularly at higher SNP densities (such as 5K and 10K), exhibiting greater stability and accuracy. This can be attributed to the unique algorithm of FarmCPU, which employs a multi-locus model that accounts for both fixed and random effects, including the kinship matrix [15,16]. This approach allows for better control of the population structure and genetic relatedness, thereby improving the SNP detection accuracy. In contrast, GEMMA uses a simpler mixed linear model (LMM) that addresses the population structure, but may not account for other confounding factors as effectively. GLM, while commonly used in genetic studies, only incorporates fixed effects and ignores random effects and genetic background, which likely contributes to its inferior performance in estimating genetic effects for complex traits. These differences in the algorithm design directly influence the stability and accuracy of genetic effect estimates, thus affecting the final selection outcomes [17].

FarmCPU’s multi-locus approach is a key distinction from the single-locus models used by GEMMA and GLM [18]. By considering multiple genetic variants simultaneously, FarmCPU can capture the complex interactions between SNPs, leading to more robust genetic predictions. This is particularly beneficial for traits controlled by multiple genes, as seen in aquaculture species like common carp, where complex genetic architectures are common.

### 3.2. The Impact of SNP Density on Genetic Prediction Accuracy

The impact of the SNP density on the genetic prediction accuracy was significantly demonstrated in this study. At lower SNP densities (e.g., 500 SNPs), the performance of GEMMA and GLM was more variable, while FarmCPU maintained higher stability and accuracy at higher SNP densities. This aligns with previous studies, which show that higher SNP densities improve the trait association and prediction accuracy by providing more genetic markers, thereby enhancing the efficiency of both GWAS and genomic selection models. In contrast, lower SNP densities offer fewer markers, which cannot fully capture genetic variation, leading to inaccurate genetic effect estimates and affecting the reliability of the predictions. The stable performance of FarmCPU at higher SNP densities further validates the critical role of the SNP density in ensuring the accuracy of genomic selection.

### 3.3. Environmental and Seasonal Effects on Model Performance

This study also revealed the impact of seasonal variation on the performance of different methods. For instance, FarmCPU showed a stronger genetic prediction accuracy for the fat content of the fillet (FC) and condition factor (CF) traits in the autumn, while GEMMA and GLM demonstrated more consistent predictions for the body length (SL) and body weight (Weight) across both spring and autumn. These seasonal differences suggest that environmental factors, such as the temperature and feeding management, may influence the genetic expression of certain traits. FarmCPU’s stable performance and higher accuracy in both spring and autumn indicate that it is better able to handle these seasonal environmental variations, providing more reliable genetic predictions.

The advantage of FarmCPU across different seasons may stem from its ability to better capture the interaction between genetic and environmental factors, particularly in multi-locus models. This also explains why FarmCPU performed better in traits with significant environmental fluctuations, such as the fat content of the fillet and condition factor. In contrast, GEMMA and GLM showed more variability in their performance across seasonal changes, likely because they did not fully account for the potential impact of environmental factors on trait genetic expression. This highlights the need for more refined models that integrate environmental data with genetic information, particularly for aquaculture species where environmental factors have a significant influence on trait expression.

### 3.4. Implications of Results for Genomic Selection in Carp

In this study, we employed multiple genomic selection (GS) models in combination with different genome-wide association study (GWAS) methods to systematically evaluate key traits in common carp. By using the top 5000 significant SNPs, we compared the performance of three widely used GWAS methods—FarmCPU, GLM, and GEMMA—within various GS frameworks. The aim was to explore their potential for improving the genomic prediction in aquaculture. Our results demonstrated clear differences among the methods in terms of SNP effect detection, prediction accuracy, and practical applicability, highlighting the importance of method selection in trait-specific genomic selection strategies.

FarmCPU consistently exhibited a superior stability and predictive performance across most traits, especially when applied to high-density SNP data. Its iterative modeling of fixed and random effects effectively controls for false positives and enhances the detection of small-to-moderate effect loci [19]. This makes FarmCPU particularly suitable for complex quantitative traits such as the body length, weight, and fat content in common carp, providing a solid foundation for GS in aquaculture breeding.

In contrast, GLM and GEMMA demonstrated greater flexibility, particularly with medium- and low-density SNP datasets (1K–5K) or large sample sizes under resource-limited scenarios. GEMMA, as a representative mixed linear model, efficiently accounts for the population structure and relatedness, providing robust results even when its prediction accuracy is slightly lower than that of FarmCPU [20]. GLM, while computationally efficient for the rapid screening of potential loci, tends to generate more false positives and should be used with caution [21].

Consistent with the findings of Palaiokostas et al. (2016), who evaluated the potential of SNPs generated via 2b-RAD sequencing for estimating breeding values related to disease resistance in common carp, our study also supports the use of GWAS-informed SNP selection to enhance the GS performance. In their study, genomic prediction models such as GBLUP and BayesB achieved higher accuracies (r = 0.38–0.46) compared to the traditional pedigree-based BLUP method (r = 0.30), highlighting the advantages of genomic information over pedigree records [22]. Similarly, Zheng et al. (2016) identified SNPs associated with fat-related traits in carp through GWAS and validated them using gene expression analysis, further reinforcing the effectiveness of integrating GWAS into GS pipelines for uncovering the genetic basis of complex traits [13].

Our study also highlights that integrating multiple GWAS methods into GS models not only improves the prediction accuracy, but also maintains genetic diversity within the population, reducing potential biases caused by reliance on a single model [23,24]. This approach is particularly advantageous for traits such as disease resistance and environmental adaptability, which are often polygenic and strongly influenced by environmental variation.

In summary, our study underscores the potential of GWAS-assisted genomic selection in common carp breeding. It emphasizes the importance of tailoring analytical strategies to specific trait architectures. Future research should aim to increase sample sizes and incorporate multi-omics data, such as transcriptomics and epigenomics, to build more comprehensive and accurate genomic prediction models that can further advance precision breeding in aquaculture species.

## 4. Materials and Methods

### 4.1. Source of Fish and Phenotypic Collection

A commercial broodstock population of mirror carp was established at the Hulan Breeding Station, Heilongjiang River Fisheries Research Institute, Chinese Academy of Fishery Sciences, located in Harbin, Heilongjiang Province, China. The eggs were randomly allocated and incubated in standardized carp hatching tanks. Following hatching, a total of 3000 larvae were transferred to a 0.1-hectare earthen pond with water depths ranging from 0.7 m to 1.2 m. At approximately 60 days post-hatching, 1000 individuals were implanted with passive integrated transponder (PIT) tags (size: 1.35 mm × 8 mm) for identification purposes and subsequently reared under controlled conditions until they reached the age of two years. One-third of these individuals were randomly selected for a phenotypic evaluation. Prior to measurement, the fish were anesthetized using 0.5 mg/L 2-phenoxyethanol (C_8_H_10_O_2_) to prevent injury during handling and cutting for some caudal fins. Each fish was assessed for the fillet fat content using a Fish Fatmeter (Distell Company, Old Levenseat, Scotland, UK) (Model FFM-692), body weight with electronic scales (accuracy: 0.1 g), and body length utilizing Vernier calipers (accuracy: 0.1 cm). The fin rays were excised and subsequently preserved in absolute ethanol during the spring phenotypic evaluation. They were then stored at −20 °C in a refrigerator until genomic DNA extraction was conducted. The experimental subjects then had measurements taken during both spring and autumn across three traits: the body length, body weight, and fat content of the fillet at 2 years old. After excluding outliers or individuals with failed measurements, a total of 325 individual phenotypic data points were ultimately obtained. Additionally, a fourth trait, the condition factor (CF), was calculated using the formula $CF=\frac{W}{L^{3}}\times 100$, where W represents the weight and L represents the length. Thus, a total of eight phenotypic traits were analyzed, including data from both seasons. The visualization of the phenotypic distributions was performed in R (version 4.4.0) and a correlation analysis was conducted across the eight traits [25].

### 4.2. Genotyping and SNP Calling

The total genomic DNA from the experimental individuals was extracted using a standard phenol–chloroform protocol. The quality of the extracted DNA was assessed using a NanoDrop 2000C spectrophotometer (Thermo Scientific, Waltham, MA, USA) and subsequently utilized for library construction. Based on the BGI T7 sequencing platform, by employing the paired-end sequencing (Paired-End) approach with a sequencing strategy of PE150 and a library size of 350 bp, the genome resequencing (10X) of 325 common carp samples was accomplished. Each sample yielded 17 Gb of raw data, generating a total of no less than 5542 Gb of raw data. The data volume of each sample was not less than 85% of the committed data volume, with an average Q30 of a data quality ≥88% and an average effective conversion rate ≥95%. Single-nucleotide polymorphisms (SNPs) were identified using bcftools (version 1.10.2) [26]. Sequencing data were aligned to the carp reference genome using BWA (version 0.7.17), and BAM files were generated. These files were then processed by samtools (version 1.10) sort for sorting and samtools markdup for duplicate removal [27,28,29]. SNP candidate sites were called using bcftools (version 1.10.2) mpileup with a minimum mapping quality of -q 20 and a minimum base quality of -Q 20. SNP calling was performed with the bcftools call in the multi-allelic mode (-m), and the -v snps parameter was used to retain only SNPs, outputting a VCF file. Low-quality SNPs were filtered using a bcftools filter, retaining sites with a quality score (QUAL) > 20, sequencing depth (DP) > 10, and mapping quality (MQ) > 20. The filtered VCF file was compressed using bcftools view, and an index was created with a bcftools index [30]. For subsequent GWAS and GS analyses, the resulting VCF file was further filtered using PLINK (version 1.90b7.2), removing sites with a genotype missing rate > 20% and a minor allele frequency (MAF) < 0.05 [31].

### 4.3. Heritability Analysis

Using the fastGWA-REML module in GCTA, the analysis was performed on eight phenotypic traits to estimate the heritability and genetic components [32]. The heritability was calculated using the following formula:

(1) $$h^{2}=\frac{\sigma_{g}^{2}}{\sigma_{g}^{2}+\sigma_{e}^{2}},$$

where $h^{2}$ represents the heritability, $\sigma_{g}^{2}$ is the genetic variance, and $\sigma_{e}^{2}$ is the environmental variance [33].

### 4.4. GWAS Analysis

Genome-wide association studies (GWAS) were performed on eight phenotypic traits of carp using three models: the mixed linear model (LMM) in GEMMA, the FarmCPU model from the RMVP package (version 4.4.0), and the general linear model (GLM) [18,34]. The GEMMA (Genome-wide Efficient Mixed Model Association) (version 0.98.6) employs a mixed linear model (LMM) for GWAS analysis. By incorporating random effects (via a kinship matrix), it corrects for the population structure and genetic relatedness between individuals, thereby reducing the false positive rate [18,35]. The LMM assumes that the phenotype is determined by both fixed effects (SNPs and covariates) and random effects (genetic background defined by the kinship matrix) [8,36].

FarmCPU (Fixed and Random Model Circulating Probability Unification) is a multi-locus GWAS model that combines the strengths of both fixed and random effects, enhancing the detection power and computational efficiency [37]. FarmCPU iteratively selects significant SNPs as covariates, progressively removing the effects of multicollinearity, making it particularly suitable for detecting complex quantitative traits controlled by multiple genes, such as those in carp [16].

The GLM (General Linear Model) is a single-locus GWAS model that assumes the phenotype is determined by the fixed effects of SNPs and covariates, without accounting for random effects such as genetic relatedness. The GLM tests the association between each SNP and the phenotype individually using linear regression, calculating *p*-values and effect sizes [38].

### 4.5. Genomic Selection (GS) Analysis

The genomic selection (GS) for the carp phenotype was performed using six machine learning methods from the Python package sklearn (version 1.3.0) and five Bayesian methods from the BGLR package in R (version 1.1.0) [39]. In the linear model (GS), ordinary least squares regression (Linear Regression) estimates SNP effects by minimizing the residual sum of squares and fitting a linear model $y=\beta_{0}+\beta_{1}x_{1}+\cdots +\beta_{n}x_{n}+\epsilon$ to predict the phenotype [40,41]. Partial least squares regression (PLS Regression) projects high-dimensional SNP data and phenotypes into a lower-dimensional latent component space, maximizing covariance, and is suitable for handling multicollinearity [42,43]. Ridge regression (Ridge) introduces an L2 regularization term, shrinking SNP effects to improve the model stability [44]. Kernel ridge regression (KernelRidge) maps SNP data to a high-dimensional space using kernel functions, capturing nonlinear genetic effects while incorporating L2 regularization [45,46]. Support vector regression with a linear kernel (SVR-linear) seeks the linear prediction function that minimizes the complexity and error, enhancing robustness against noise. Support vector regression with a polynomial kernel (SVR-poly) models the nonlinear SNP–phenotype relationship using a polynomial kernel, suitable for complex genetic architectures [47]. BayesA assumes that SNP effects follow a t-distribution, allowing for different variances across markers to capture heterogeneous effects. BayesB introduces a sparsity assumption (some SNP effects are zero), making it suitable for scenarios with few large-effect QTLs. BayesC applies a mixed distribution (zero and non-zero effects) to enhance the sparsity. BayesLASSO (BL) applies L1 regularization using a Laplace prior to this to shrink small effects and retain large ones [48,49]. Bayesian Ridge Regression (BRR) uses a normal one prior to this and behaves similarly to ridge regression, balancing the shrinkage of all SNP effects [50].

For the GS analysis, the models were evaluated using five-fold cross-validation, repeated ten times, resulting in 50 test runs per model. The SNPs used for the analysis were selected using various GWAS models based on *p*-values, with the top 500, 1000, 5000, 10,000, and 50,000 SNPs selected in separate gradients. Finally, results were visualized using the ggplot2 package in R [25,51].

## 5. Conclusions

This study evaluated the performance of three GWAS methods (FarmCPU, GEMMA, and GLM) combined with 11 genomic selection (GS) models for common carp growth traits across spring and autumn seasons. The results show that FarmCPU outperforms the GEMMA and GLM in terms of its stability and predictive accuracy, particularly under higher SNP densities (e.g., 5K), where the prediction accuracy exceeds 0.8 for most traits. The combination with Bayesian methods yields the best performance, with the prediction accuracy for the FC_S trait surpassing 0.95. Furthermore, the integration of FarmCPU with GS models provided the most reliable and efficient genomic selection approach, especially for traits with complex genetic architectures. These findings highlight the significant potential of combining GWAS and GS methods for the genetic improvement of aquaculture species. This study also underscores the importance of optimizing the SNP density and developing refined models to account for seasonal variations, which are crucial for enhancing the effectiveness and sustainability of breeding programs in aquaculture.

## Figures and Tables

**Table 1:** Results of fastGWA_REML for each phenotype, including heritability, *p*-values for heritability (indicating the significance of the genetic influence on the phenotype), genetic variance, and environmental variance.

Trait	Heritability	Pval	Vg	Ve
SL_S	0.3367	0.0111	2.7868 ± 1.0972	5.4904 ± 0.8285
Weight_S	0.3145	0.0133	7020.45 ± 2836.48	15,299.9 ± 2204.44
FC_S	0.0000	1.0000	3.4787 × 10^−18^ ± 0.0054	0.0564 ± 0.0061
CF_S	0.3196	0.0257	0.0311 ± 0.0139	0.0662 ± 0.0105
SL_F	0.5298	0.0005	5.8179 ± 1.6799	5.164 ± 1.0661
Weight_F	0.3554	0.0089	27,012 ± 10,329	48,996.1 ± 7624.17
FC_F	0.5426	0.0004	0.4877 ± 0.1368	0.4111 ± 0.0848
CF_F	0.0234	0.7751	0.0066 ± 0.0233	0.2788 ± 0.0291

## Data Availability

The raw sequence data reported in this paper have been deposited in the Genome Sequence Archive of the National Genomics Data Center, Beijing Institute of Genomics, Chinese Academy of Sciences (accession number: PRJCA037933).
